# The knowing‐doing gap in acute stroke—Does stroke knowledge translate into action?

**DOI:** 10.1002/brb3.1245

**Published:** 2019-02-20

**Authors:** Kashif W. Faiz, Antje Sundseth, Bente Thommessen, Ole M. Rønning

**Affiliations:** ^1^ Department of Neurology Akershus University Hospital Lørenskog Norway; ^2^ Health Services Research Unit Akershus University Hospital Lørenskog Norway; ^3^ Institute of Clinical Medicine University of Oslo Oslo Norway

**Keywords:** knowledge, prehospital delay, risk factors, stroke, thrombolysis

## Abstract

In this study, we suggest that there is a substantial knowing‐doing gap in acute stroke, as increased stroke knowledge was not associated with earlier hospital admission.

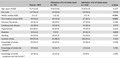

## 1 INTRODUCTION

More than two decades after the first study on intravenous thrombolysis in acute ischemic stroke was published (The National Institute of Neurological Disorders and Stroke rt‐PA Stroke Study Group, 1995); the utilization rates still are low (Bouckaert, Lemmens, & Thijs, 2009; Teuschl & Brainin, 2010). Time plays an important role in acute stroke management, and several attempts have been made in order to reduce the time from symptom onset to hospital arrival, often referred to as prehospital delay (Teuschl & Brainin, 2010).

Educational campaigns, acronyms, and different types of stroke codes and fast tracks have been introduced, with mixed degree of success (Lecouturier et al., 2010). Important obstacles to minimize the prehospital delay are insufficient stroke knowledge, not attributing symptoms to stroke, and, even more importantly, not being able to translate knowledge into action (Jones, Jenkinson, Leathley, & Watkins, 2010). Factors influencing decisions to seek immediate hospitalization seem to be complex (Mandelzweig, Goldbourt, Boyko, & Tanne, 2006).

Few studies have compared stroke knowledge directly with prehospital delay. In this study, we aimed to assess if knowledge of stroke symptoms and risk factors was associated with early hospital admission, that is, to determine if a knowing‐doing gap exists in acute stroke.

## 2 METHODS

In this cross‐sectional study, we included consecutive stroke patients admitted to the Stroke Unit, Department of Neurology, Akershus University Hospital, during a 1‐year period (April 2009–March 2010). Knowledge of stroke symptoms and risk factors was explored by using an open‐ended questionnaire. Patients were approached by KWF or AS within 72 hr after admission, and information was obtained by interviewing the patients and by medical record review (including medication use). The diagnosis of stroke was made by the treating neurologist according to the World Health Organization definition. Patients with stroke mimics, transient ischemic attack, and patients unable to answer the questions were excluded. Only patients aged ≥18 years were included in the present study. In all, 340 stroke patients were approached, of whom 207 (60.9%) were able to answer the questions.

Knowledge of stroke symptoms was defined as identifying at least two of the three FAST‐symptoms (facial drop, numbness/weakness of the arm/leg, and speech problems). Knowledge of stroke risk factors was defined as identifying at least two of the three modifiable and common risk factors hypertension, smoking, and diabetes. Early admission was defined as admission within 4 hr after symptom onset.

Categorical variables are presented as frequencies and percentages, and continuous variables as means and standard deviations for normally distributed data, and medians and interquartile ranges for nonparametric data. Between‐group differences for categorical variables were determined by chi‐squared statistics or Fisher exact test, as appropriate (categorical variables), and by unpaired two‐sample *t* test and Mann–Whitney *U* test for continuous variables (normally distributed and nonparametric variables, respectively).

Multivariate logistic regression analyses were performed to identify factors related to early admission. Three models for stroke knowledge were used: (a) knowledge of stroke symptoms, (b) knowledge of stroke risk factors, and (c) knowledge of stroke symptoms *and* risk factors.

The study was approved by the Regional Ethics Committee (REC) and the hospital's Data Protection Official. All participants gave their oral, informed consent, in line with recommendations from the REC.

## 3 RESULTS

A total of 207 patients were included in the study, with a mean age of 71.9 years (*SD* = 12.0) and median National Institutes of Health Stroke Scale (NIHSS) score of 3 (interquartile range 1–5). Table 1 shows the clinical characteristics of the study population. Ninety‐five patients (46.0%) were admitted within 4 hr of symptom onset, and these patients had more severe strokes (*p *< 0.001), a higher proportion contacted the Emergency Medical Services (EMS) as the first medical contact (*p *< 0.001), and fewer had diabetes (*p *= 0.013). There were no differences regarding knowledge of stroke symptoms (*p *= 0.298), knowledge of stroke risk factors (*p *= 0.781), or knowledge of stroke symptoms and risk factors (*p *= 0.389) between patients with early and late admission.

**Table 1:** Patient characteristics

	Total (*n* = 207)	Admission ≤4 hr of stroke onset (*n* = 95)	Admission >4 hr of stroke onset (*n* = 112)	*p* Value
Age, years, *M* (SD)	71.9 (12.0)	70.8 (13.0)	72.8 (11.0)	0.237
Sex, male	117 (56.5)	54 (56.8)	63 (56.3)	0.932
NIHSS, median (IQR)	3 (1–5)	4 (2–8)	3 (1–4)	**<0.001**
First medical contact EMS	96 (46.4)	69 (72.6)	27 (24.1)	**<0.001**
Previous TIA/stroke	65 (31.4)	28 (29.5)	37 (33.0)	0.582
Cardiovascular disease	63 (30.4)	29 (30.5)	34 (30.4)	0.979
Hypertension	143 (69.1)	64 (67.4)	79 (70.5)	0.623
Diabetes	34 (16.4)	9 (9.5)	25 (22.3)	**0.013**
Smoking	53 (25.6)	20 (21.1)	33 (29.5)	0.167
Ischemic stroke	190 (91.8)	84 (88.4)	106 (94.6)	0.104
Knowledge of stroke symptoms^a^	90 (43.5)	45 (47.4)	45 (40.2)	0.298
Knowledge of stroke risk factors^b^	29 (14.0)	14 (14.7)	15 (13.4)	0.781
Knowledge of stroke symptoms and risk factors^a^^,^ b	22 (10.6)	12 (12.6)	10 (8.9)	0.389

*Note.*

Categorical variables are presented as frequencies and percentages. Age presented as mean and standard deviation, and NIHSS as median and interquartile range (IQR). Significant values were in bold.

EMS: Emergency Medical Services; NIHSS: National Institutes of Health Stroke Scale; TIA: Transient Ischemic Attack.

^a^Able to identify ≥2 of the three symptoms facial drop, numbness or weakness of the arm and/or leg, and speech problems. ^b^Able to identify ≥2 of the three risk factors hypertension, smoking, and diabetes.

Prehospital delay was not associated with neither knowledge of stroke symptoms (*p *= 0.066) nor risk factors (*p *= 0.791) (Table 2).

**Table 2:** Knowledge of stroke symptoms and risk factors (*n* = 201)

Number of stroke symptoms/risk factors identified^a^^,^ b	Stroke symptoms knowledge (%)^a^	Prehospital delay, median (IQR)	Stroke risk factors knowledge (%)^b^	Prehospital delay, median (IQR)
0/3	70 (33.8)	3.8 (1.4–12.1)	141 (68.1)	4.8 (1.6–13.7)
1/3	47 (22.7)	8.4 (2.2–22.2)	37 (17.9)	4.0 (1.5–14.6)
2/3	63 (30.4)	4.1 (1.4–12.7)	26 (12.6)	4.5 (1.9–10.8)
3/3	27 (13.0)	3.4 (2.0–7.4)	3 (1.4)	2.9 (0.9–7.4)
*p* Value (Kruskall–Wallis test)		0.066		0.791

*Note*

IQR: interquartile range.

^a^Stroke symptoms: facial drop, numbness or weakness of the arm and/or leg, and speech problems. ^b^Stroke risk factors: hypertension, smoking, and diabetes.

In all multivariate models, only NIHSS on admission and EMS as the first medical contact were significantly associated with early admission, and there was a trend toward younger age (*p*‐values between 0.056 and 0.082). Stroke knowledge was not associated with early hospital admission in any of the multivariate models (Table 3).

**Table 3:** Multivariate analyses on early admission (admission ≤ 4 hr of stroke onset)

	*p* Value Model 1^a^	*p* Value Model 2^b^	*p* Value Model 3^c^
Age	0.082	0.056	0.072
Sex	0.535	0.475	0.469
Previous stroke/TIA	0.202	0.191	0.177
Risk factors^d^	0.474	0.453	0.485
NIHSS on admission	0.003	0.003	0.003
EMS as first medical contact	<0.001	<0.001	<0.001
Knowledge	0.287	0.621	0.470

*Note*

EMS: Emergency Medical Services; NIHSS: National Institutes of Health Stroke Scale; TIA: Transient Ischemic Attack.

^a^Knowledge of stroke symptoms. ^b^Knowledge of stroke risk factors. ^c^Knowledge of stroke symptoms *and* risk factors. ^d^At least two stroke risk factors from: hypertension, smoking, diabetes, hyperlipidemia, and atrial fibrillation.

## 4 DISCUSSION

The present study confirmed that there is a substantial knowing‐doing gap in acute stroke, as increased knowledge was not associated with earlier hospital admission. In multivariate models, only severe symptoms and EMS as the first medical contact were associated with early admission.

Previous studies on stroke knowledge, both in acute stroke patients and in the general population, conclude that the level of knowledge is low or suboptimal. Importantly, our study shows in addition an inability to rapidly translate knowledge into action on symptom onset.

Because of an aging and growing population, it is assumed that the number of stroke victims will increase considerably over the next decades. Better stroke knowledge will likely be beneficial in improving rapid response at symptom onset, so that a higher proportion of patients can utilize the effective but time‐sensitive treatment options. Unfortunately, the results from this study indicate that there is a discrepancy between theoretical knowledge of stroke symptoms and risk factors, and the ability to act properly in a real situation.

Our results are in line with the few previous studies who have related stroke knowledge directly to prehospital delay (Cheung, 2001; Williams, Bruno, Rouch, & Marriott, 1997), none being able to show that knowledge was associated with shorter delay. In parallel, population‐based studies also show that knowledge is not associated with the intent to call the EMS (Fussman, Rafferty, Lyon‐Callo, Morgenstern, & Reeves, 2010). As symptom severity expressed as higher NIHSS scores is associated with shorter prehospital delay, it is plausible to ask whether mild/moderate and atypical symptoms in stroke are undercommunicated in public campaigns and information given to patients and caregivers.

There is no common agreement on how to define and assess stroke knowledge, that is, which and how many symptoms and risk factors should be included. In addition, the methods used will always influence the results; in this study, we used an open‐ended questionnaire, which often underestimate the “true knowledge,” while close‐ended questions overestimate it.

The present study has several limitations. It is a small, single‐center study. Importantly, not all stroke patients were able to answer the questionnaire in the acute setting because of their symptoms; hence, there may be a selection bias as the patients included in the study have lower median NIHSS than would be expected in an unselected acute stroke population. In addition, knowledge bias could exist, as patients could name their own symptoms as stroke symptoms.

How can we fill this knowing‐doing gap? Traditional information campaigns tend to have varying and short‐term effect. Not only more focus on time sensitivity and lack of pain in the majority of patients is necessary but also to emphasize that a proportion of patients have mild/moderate or atypical symptoms, and that time is of importance also for these patients. In addition, behavioral and perceptual factors are involved (Zock, Kerkhoff, Kleyweg, & van de Beek, 2016), and elements from change management and change theory should be considered when designing new campaigns, so that more patients can translate stroke knowledge into action. In addition, larger studies to adequately address the association between patients’ knowledge and their actions are warranted.

## CONFLICT OF INTEREST

None declared.
